# A 9 mRNAs-based diagnostic signature for rheumatoid arthritis by integrating bioinformatic analysis and machine-learning

**DOI:** 10.1186/s13018-020-02180-w

**Published:** 2021-01-11

**Authors:** Jianyong Liu, Ningjie Chen

**Affiliations:** 1 The First Department of Joint Surgery, Weifang People’s Hospital Shandong Province (The First Affiliated Hospital of Weifang University), Weifang, 261041 Shandong China; 2 The Department of Joint Surgery, Zibo Central Hospital, Shandong University, No 54 Gongqingtuan West Road, Zibo, 255036 Shandong China

**Keywords:** Rheumatoid arthritis, Diagnostic signature, Differentially expressed genes, Bioinformatics analysis, Random forest model

## Abstract

**Background:**

Rheumatoid arthritis (RA) is an autoimmune rheumatic disease that carries a substantial burden for both patients and society. Early diagnosis of RA is essential to prevent disease progression and select an optimal therapeutic strategy. However, RA diagnosis is challenging, partly due to a lack of reliable biomarkers. Here, we aimed to explore the diagnostic signature and establish a predictive model of RA.

**Methods:**

The mRNA expression profiling data of GSE17755, containing blood samples of 112 RA patients and 53 healthy control patients, were obtained from the Gene Expression Omnibus (GEO) database, followed by differential expression, GO (Gene Ontology), and KEGG (Kyoto Encyclopedia of Genes and Genomes) enrichment analysis. A PPI network was constructed to select candidate hub genes, then logistic regression and random forest models were established based on the identified genes.

**Results:**

Significantly, we identified 52 differentially expressed genes (DEGs), including 16 upregulated genes and 36 downregulated genes in RA samples compared with control samples. GO and KEGG analysis showed that several immune-related cellular processes were particularly enriched. We identified nine hub genes in the PPI network, including CFL1, COTL1, ACTG1, PFN1, LCP1, LCK, HLA-E, FYN, and HLA-DRA. The logistic regression and random forest models based on the nine identified genes reliably distinguished the RA samples from the healthy samples with substantially high AUC.

**Conclusion:**

The diagnostic logistic regression and random forest models based on nine hub genes reliably predicted the occurrence of RA. Our findings could provide new insights into RA diagnostics.

**Supplementary Information:**

The online version contains supplementary material available at 10.1186/s13018-020-02180-w.

## Introduction

Rheumatoid arthritis (RA) is an autoimmune rheumatic inflammatory disorder that influences several organs and tissues and causes chronic synovial inflammation, ultimately resulting in chronic disability, joint destruction, and decreased life expectancy [1–3]. RA affects nearly 0.5 to 1% of people globally, occurring more commonly in females [4]. Furthermore, RA is challenging to manage and often requires lifelong treatment once developed [5]. Detection of RA at an early stage affords a window of opportunity for effective curative responses, and this pre-clinical period may be as short as several months [6–8]. Accordingly, early diagnosis of RA is essential to prevent the progression of radiologic variations and select the optimal therapeutic strategy [9].

Rheumatoid factor (RF) serum biomarkers have been used as preferred diagnostic criteria for RA for decades of years [10]. However, because of the lack of sensitivity (50–90%) and specificity (50–95%) [11] of auxiliary biomarkers, anti-citrullinated protein antibody (ACPA) was included in the diagnostic criteria for RA as developed by the American College of Rheumatology (ACR)/European League Against Rheumatism (EULAR) in 2010 [12]. Existing biomarkers may be difficult to detect during the pre-clinical period. Subsequently, multiple studies have revealed an association between genetics and RA [13, 14], indicating that aberrantly expressed genes may be identified as potential diagnostic biomarkers of RA. A previous study demonstrated that dysregulated circular RNAs in the peripheral blood mononuclear cells of RA patients presented diagnostic value [15]. Multiple microRNAs have been identified as effective markers for RA patients [16]. However, the development of RA is a complex process, making it particularly important to establish a diagnostic model.

In this study, we aimed to identify blood-derived mRNA-based diagnostic signatures by integrating bioinformatics analysis and machine learning algorithms based on the mRNA expression profiling data of GSE17755 from the GEO database, containing blood samples of 112 RA patients and 53 healthy control patients. We identified a total of 52 differential expression genes (DEGs) in the RA patients compared with the controls and identified nine hub genes, including CFL1, COTL1, ACTG1, PFN1, LCP1, LCK, HLA-E, FYN, and HLA-DRA. The logistic regression and random forest models based on these nine genes reliably distinguished the RA samples from the healthy control samples.

## Materials and methods

### Data collection

To establish the diagnosis model of RA from blood sample, the mRNA expression profiling data of GSE17755 contained blood samples of 112 RA patients and 53 healthy controls were obtained from GEO (https://www.ncbi.nlm.nih.gov/geo/) [17]. The mRNA expression levels of the GSE17755 data set were quantified based on the Hitachisoft AceGene Human Oligo Chip 30K 1 Chip Version.

### Identification of differentially expressed genes (DEGs)

The dataset of GSE17755 was normalized by robust multi-array (RMA) and the DEGs were analyzed by using a *limma R* package [18]. After quantile normalization, raw signals of analyses were log2 transformed. DEGs were defined by absolute value of fold change (FC) > 2 (|log2FC| > 1) and false discovery rate (FDR) < 0.05.

### Gene Ontology (GO) and Kyoto Encyclopedia of Genes and Genomes (KEGG) analysis

To analyze the functions and pathways associated with DEGs, data were merged to obtain gene symbols, then GO enrichment analysis and KEGG pathway analysis were performed by using *enrichGO* function and *enrichKEGG* function of *clusterProfiler* package of *R* [19], respectively. Subsequently, GO enrichment results were visualized by using a *GOChord* function in *GOplot* package [20], and KEGG enrichment results were visualized by using a *Barplot* function in *clusterProfiler* package, independently. The GO included molecular function, biological process, and cellular component. The *P* < 0.05 was regarded as statistically significant.

### PPI analysis

The protein-protein analysis (PPI) was conducted in the Search Tool for the Retrieval of Interacting Genes/Proteins (STRING) database (https://string-db.org/cgi/input.pl) with the threshold of confidence score ≥ 0.4 [21]. The visualization of the PPI network was presented by Cytoscape software (https://cytoscape.org/) [22]. The modular analysis of the PPI network using the molecular complex detection (MCODE) plug-in of Cytoscape software with MCODE score > 2 as the threshold [23].

### Construction of logistic regression and random forest model

The logistic regression model and random forest model were established based on the identified genes in the PPI network, in which the expression of identified DEGs served as continuous variable, and the sample type (RA or not) served as a binary responsive variable. The logistic regression model was constructed using *glm* of R [24]. The random forest model based on the Bagging method was constructed using *randomForest R* package [25]. The 5-fold cross-validation was performed in the models using *caret R* package (https://CRAN.R-project.org/package=caret). The receiver operating characteristic curves were generated to evaluate the sensitivity and specificity of the models, and the area under the curve (AUC) was calculated to assess the reliability of the models.

## Results

### Identification of DEGs

**Fig. 1:**
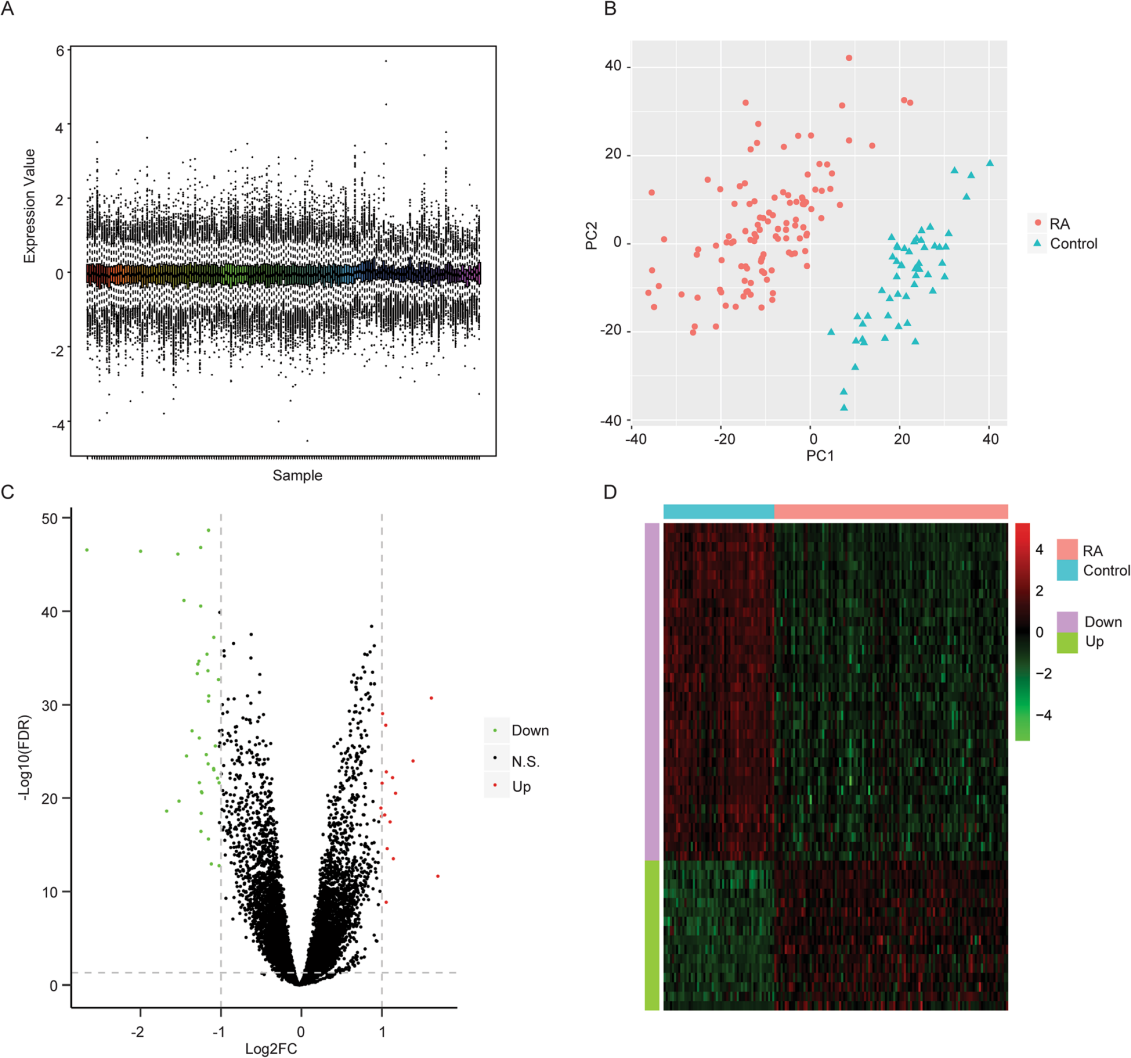
Identification of DEGs. **a** The dataset of GSE17755 was normalized by robust multi-array (RMA) and the result was shown in the box-plot. The *x*-axis was the samples and the *y*-axis was the gene expression levels. **b** The principal component analysis (PCA) based on the mRNA expression value of the samples was performed, in which the dots with different colors represented samples in different groups. The distance of the dots represented the similarity of mRNA expression of the samples. **c** Volcano plot filtering map displayed DEGs in the RA samples compared with the normal samples. The *x*-axis was the Log2fold change (FC) and the *y*-axis was −log10 (FDR). **d** The DEGs were presented by heatmap. The *x*-axis was samples and the *y*-axis was DEGs, in which red and green represented the expression level of genes, respectively

To comprehensively understand the development of rheumatoid arthritis (RA) and explore the potential diagnostic biomarkers, the mRNA expression profiling data of GSE17755, containing blood samples of 112 RA patients and 53 healthy controls, were obtained from GEO database. The dataset was normalized by robust multi-array (RMA), and we observed that the data deviation was acceptably small, which could be used for further analysis (Fig. 1a and Table S1). In order to verify the data repeatability, the principal component analysis (PCA) based on the mRNA expression value of the samples was performed, and our data revealed that the samples of RA patients and healthy controls were effectively separated (Fig. 1b), indicating that the availability of the data repeatability. Significantly, we identified a total of 52 DEGs, including 16 upregulated genes and 36 downregulated genes in the RA samples compared with the normal samples (Fig. 1c), in which the remarkable difference was presented by heatmap (Fig. 1d).

### GO and KEGG analysis of DEGs

**Fig. 2:**
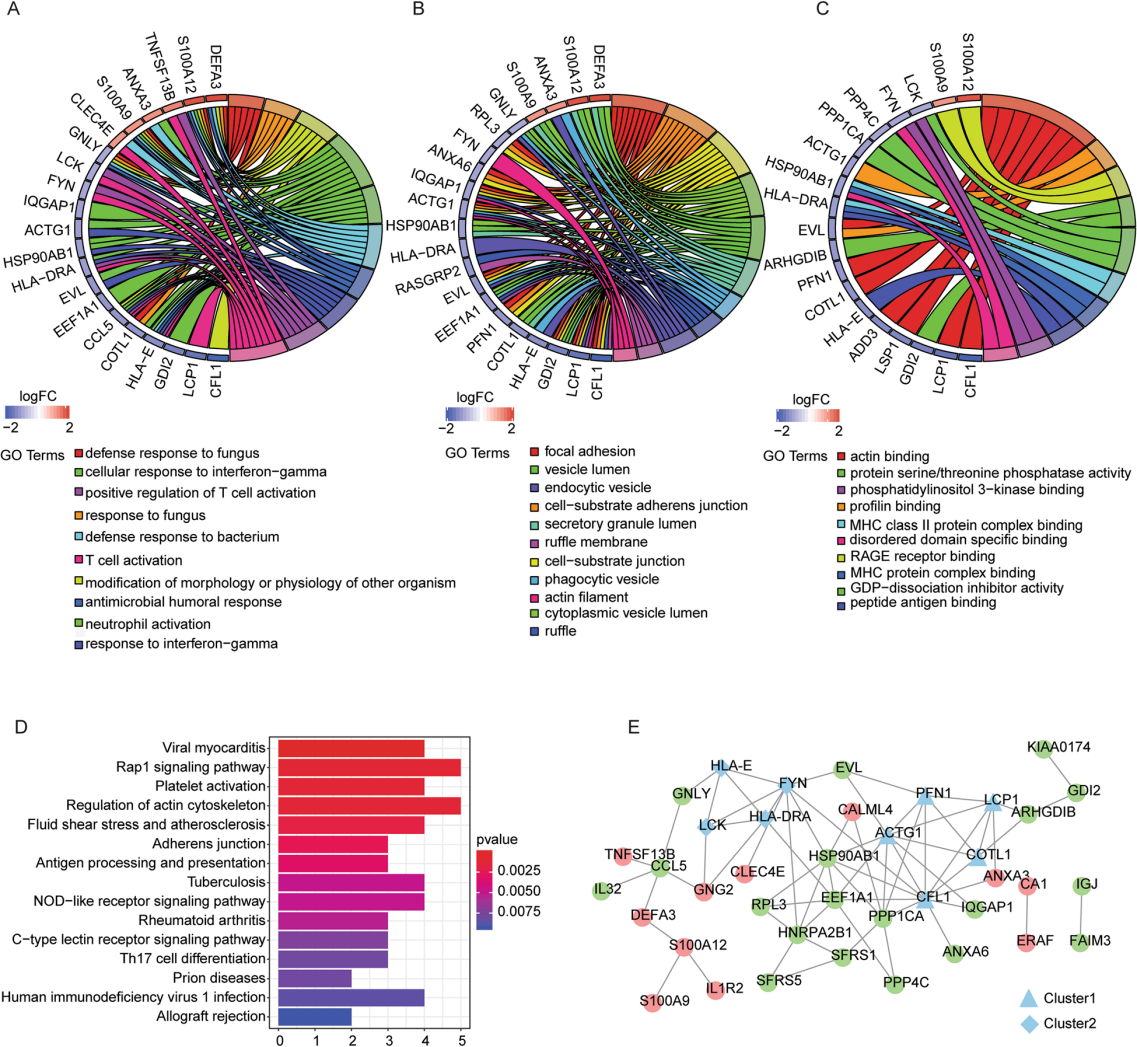
GO and KEGG analysis of DEGs and PPI network construction. The GO and KEGG analysis were performed based on the identified DEGs using *clusterProfiler* package of *R*. The top 10 significant biological process (**a**) and cellular component (**b**) GO terms, and the 11 remarkable molecular function GO terms (**c**) were demonstrated. The right half-circle was the enriched GO terms, which were presented in different colors. The left half-circle was the gene enriched in these terms, in which red represented upregulation and blue represented downregulation. **d** The top 15 notable KEGG pathways were shown in the bar plot. The *y*-axis was the name of signaling pathways and the *x*-axis was the gene number. **e** PPI network based on the identified DEGs was constructed in the STRING online database. Each dot represented a node and the number of lines connected to the dot represented the degree of the node. The red represented the upregulated genes and the green represented the downregulated genes. The blue triangle was the Cluster1 and the blue diamond was the Cluster2

For primary comprehensions of these DEGs, GO [16] and KEGG pathway analysis were performed based on the identified DEGs. We enriched 102 GO terms and 41 KEGG pathways in the analysis (*P* < 0.05) (Table S2). The top 10 significant biological process and cellular component [26] GO terms (Fig. 2a, b), the 11 remarkable molecular function GO terms (Fig. 2c), and the top 15 notable KEGG pathways were demonstrated (Fig. 2d), in which multiple cellular processes were associated with immune response.

### PPI network construction and candidate hub gene selection

To further identify the candidate hub genes among the DEGs in the healthy cases and RA patients, we constructed PPI network based on the 52 DEGs in the STRING online database (https://string-db.org/cgi/input.pl), and we identified 39 genes with confidence score ≥ 0.4 in the PPI network (Fig. 2e). The network module may represent the specific biological significance and thereby is usually the core of the PPI network [27]. Accordingly, we performed the modular analysis of the PPI network using the MCODE plug-in of Cytoscape software with MCODE score > 2 as the threshold and identified Cluster1 including CFL1, COTL1, ACTG1, PFN1, and LCP1, and Cluster2 containing LCK, HLA-E, FYN, and HLA-DRA (Fig. 2e), suggesting that these nine genes may play critical roles in the development of RA.

### Construction of logistic regression and random forest model

**Fig. 3:**
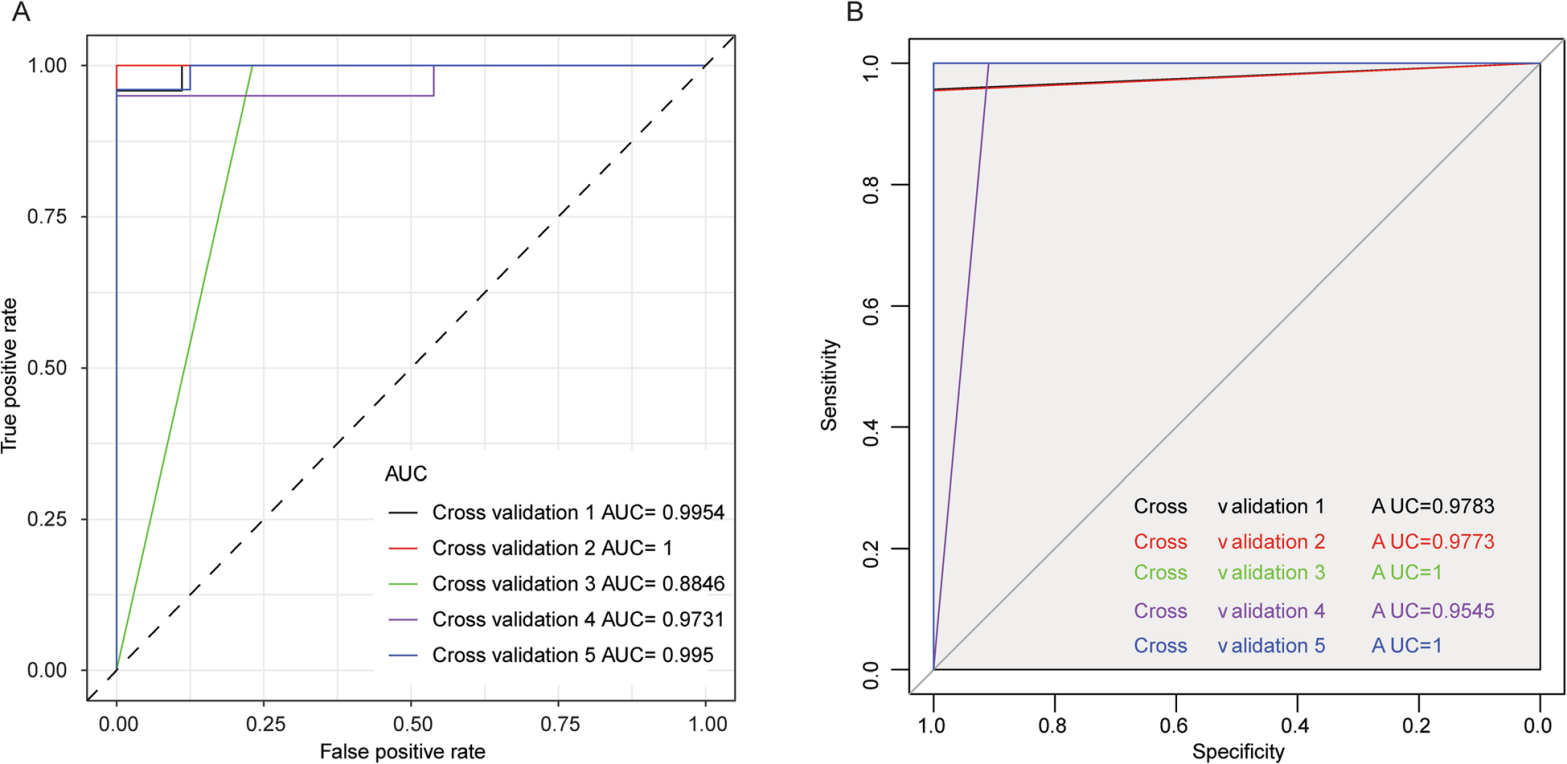
Construction of logistic regression and random forest model. **a**, **b** The logistic regression model and the random forest model based on the selected genes were constructed using *glm* of R and *randomForest R* package, respectively. The reliability of the model was assessed by the AUC analysis. **a** The 5-fold cross-validation was performed in the logistic regression model. **b** The 5-fold cross-validation was performed in the random forest model

We constructed the logistic regression model and the random forest model based on the selected nine genes including CFL1, COTL1, ACTG1, PFN1, LCP1, LCK, HLA-E, FYN, and HLA-DRA in the PPI network, in which the expression of selected nine genes served as the continuous predict variable and the sample type (RA or not) served as the response variable. The 5-fold cross-validation was performed in the model to verify the reliability of the model and we observed that the AUC of the logistic regression model (Fig. 3a) and the random forest model (Fig. 3b) was substantially high, suggesting that both models can reliably distinguish the RA samples from the healthy control samples.

## Discussion

Consistent with the results of previous studies, our study indicates that RA is a disease involving a complex gene network and multiple gene contributors [28]. In this study, 39 genes were selected in the PPI network and 9 hub genes were identified after modular analysis of the PPI network, including CFL1, COTL1, ACTG1, PFN1, LCP1, LCK, HLA-E, FYN, and HLA-DRA. These genes may be significantly correlated with the progression of RA. Furthermore, we constructed a logistic regression model and random forest model based on the nine identified genes, both with a significant AUC.

Combined with previous reports, COTL1, LCK, HLA-DRA, and HLA-E, among our identified hub genes, have been reported to be associated with RA. Proteomics revealed that upregulation of COTL1 might affect the 5-lipoxygenase (5LO) activity involved in leukotriene biosynthesis and mediate inflammation in RA [29]. Whole-exome sequencing defined LCK as linked to familial RA and highlighted LCK variation in the T cell receptor (TCR) signaling pathway leading to T cell activation, resulting in T cell differentiation, survival, and effector functions [30]. Bioinformatics analysis showed that HLA-DRA was dysregulated in RA patients [31, 32]. Furthermore, HLA-E was involved in susceptibility to RA and anti-TNF treatment in RA patients [33]. Little evidence has directly demonstrated any relation of other genes to RA, such as CFL1, ACTG1, PFN1, or FYN; however, these genes play a key role in immune regulation [34–37].

In conclusion, we selected innovative biomarkers by analyzing the critical genes that influence the molecular mechanisms of RA, and nine mRNA-based diagnostic signatures were identified. The logistic regression and random forest models based on these nine hub genes were able to reliably distinguish RA samples from healthy control samples. Meanwhile, the nine genes had immune-related functions, including T cell activation, differentiation, tolerance, and lymphocyte formation. Further exploration is warranted to validate the clinical significance of these genes in the immune disorder of RA progression.

## Supplementary Information

**Additional file 1: Table S1.** MRNA expression levels of each sample after data standardization

**Additional file 2: Table S2.** Significantly GO terms and KEGG pathways

## Data Availability

The datasets analyzed during the current study are available in the [GEO] repository, [https://www.ncbi.nlm.nih.gov/geo/].

## References

[1] Ye Z, Liang Y, Ma Y, Lin B, Cao L, Wang B (2018). Targeted photodynamic therapy of cancer using a novel gallium (III) tris (ethoxycarbonyl) corrole conjugated-mAb directed against cancer/testis antigens 83. Cancer Med.

[2] van der Woude D, van der Helm-van Mil AHM (2018). Update on the epidemiology, risk factors, and disease outcomes of rheumatoid arthritis. Best Pract Res Clin Rheumatol.

[3] Smolen JS, Aletaha D, McInnes IB (2016). Rheumatoid arthritis. Lancet.

[4] Charles J, Britt H, Pan Y (2013). Rheumatoid arthritis. Aust Fam Phys.

[5] Haro I, Sanmarti R (2013). Rheumatoid arthritis: current advances in pathogenesis, diagnosis and therapy. Curr Top Med Chem.

[6] Huizinga TW, Landewe RB (2005). Early aggressive therapy in rheumatoid arthritis: a ‘window of opportunity’?. Nat Clin Pract Rheumatol.

[7] Chaudhry M, Wilson AG (2017). The role of genetic analysis for predicting outcome of rheumatoid arthritis. Expert Rev Mol Diagn.

[8] Coffey CM, Crowson CS, Myasoedova E, Matteson EL, Davis JM (2019). Evidence of diagnostic and treatment delay in seronegative rheumatoid arthritis: missing the window of opportunity. Mayo Clin Proc.

[9] Littlejohn EA, Monrad SU (2018). Early diagnosis and treatment of rheumatoid arthritis. Prim Care.

[10] Deane KD (2018). Preclinical rheumatoid arthritis and rheumatoid arthritis prevention. Curr Rheumatol Rep.

[11] Matuszewska A, Madej M, Wiland P (2016). Immunological markers of rheumatoid arthritis. Postepy Hig Med Dosw.

[12] Aletaha D, Neogi T, Silman AJ, Funovits J, Felson DT, Bingham CO (2010). 2010 rheumatoid arthritis classification criteria: an American College of Rheumatology/European League Against Rheumatism collaborative initiative. Ann Rheum Dis.

[13] Messemaker TC, Huizinga TW, Kurreeman F (2015). Immunogenetics of rheumatoid arthritis: Understanding functional implications. J Autoimmun.

[14] Eyre S, Bowes J, Diogo D, Lee A, Barton A, Martin P (2012). High-density genetic mapping identifies new susceptibility loci for rheumatoid arthritis. Nat Genet.

[15] Yang X, Li J, Wu Y, Ni B, Zhang B (2019). Aberrant dysregulated circular RNAs in the peripheral blood mononuclear cells of patients with rheumatoid arthritis revealed by RNA sequencing: novel diagnostic markers for RA. Scand J Clin Lab Invest.

[16] Evangelatos G, Fragoulis GE, Koulouri V, Lambrou GI (2019). MicroRNAs in rheumatoid arthritis: From pathogenesis to clinical impact. Autoimmun Rev.

[17] Lee HM, Sugino H, Aoki C, Nishimoto N (2011). Underexpression of mitochondrial-DNA encoded ATP synthesis-related genes and DNA repair genes in systemic lupus erythematosus. Arthritis Res Ther.

[18] Ritchie ME, Phipson B, Wu D, Hu Y, Law CW, Shi W (2015). Limma powers differential expression analyses for RNA-sequencing and microarray studies. Nucleic Acids Res.

[19] Yu G, Wang LG, Han Y, He QY (2012). clusterProfiler: an R package for comparing biological themes among gene clusters. OMICS.

[20] Walter W, Sanchez-Cabo F, Ricote M (2015). GOplot: an R package for visually combining expression data with functional analysis. Bioinformatics.

[21] Szklarczyk D, Gable AL, Lyon D, Junge A, Wyder S, Huerta-Cepas J (2019). STRING v11: protein-protein association networks with increased coverage, supporting functional discovery in genome-wide experimental datasets. Nucleic Acids Res.

[22] Zhang C, Peng L, Zhang Y, Liu Z, Li W, Chen S (2017). The identification of key genes and pathways in hepatocellular carcinoma by bioinformatics analysis of high-throughput data. Med Oncol.

[23] Bader GD, Hogue CW (2003). An automated method for finding molecular complexes in large protein interaction networks. BMC Bioinformatics.

[24] Moutouama FT, Biaou SSH, Kyereh B, Asante WA, Natta AK (2019). Factors shaping local people's perception of ecosystem services in the Atacora Chain of Mountains, a biodiversity hotspot in northern Benin. J Ethnobiol Ethnomed.

[25] Alderden J, Pepper GA, Wilson A, Whitney JD, Richardson S, Butcher R (2018). Predicting pressure injury in critical care patients: a machine-learning model. Am J Crit Care.

[26] Atzeni F, Talotta R, Masala IF, Bongiovanni S, Boccassini L, Sarzi-Puttini P (2017). Biomarkers in Rheumatoid Arthritis. Isr Med Assoc J.

[27] Xia J, Benner MJ, Hancock RE (2014). NetworkAnalyst--integrative approaches for protein-protein interaction network analysis and visual exploration. Nucleic Acids Res.

[28] Gregersen PK (1999). Genetics of rheumatoid arthritis: confronting complexity. Arthritis Res.

[29] Jin EH, Shim SC, Kim HG, Chae SC, Chung HT (2009). Polymorphisms of COTL1 gene identified by proteomic approach and their association with autoimmune disorders. Exp Mol Med.

[30] Wang Y, Chen S, Chen J, Xie X, Gao S, Zhang C (2020). Germline genetic patterns underlying familial rheumatoid arthritis, systemic lupus erythematosus and primary Sjogren's syndrome highlight T cell-initiated autoimmunity. Ann Rheum Dis.

[31] Hao R, Du H, Guo L, Tian F, An N, Yang T (2017). Identification of dysregulated genes in rheumatoid arthritis based on bioinformatics analysis. PeerJ.

[32] Xiao X, Hao J, Wen Y, Wang W, Guo X, Zhang F (2016). Genome-wide association studies and gene expression profiles of rheumatoid arthritis: an analysis. Bone Joint Res.

[33] Iwaszko M, Swierkot J, Kolossa K, Jeka S, Wiland P, Bogunia-Kubik K (2015). Polymorphisms within the human leucocyte antigen-E gene and their associations with susceptibility to rheumatoid arthritis as well as clinical outcome of anti-tumour necrosis factor therapy. Clin Exp Immunol.

[34] Dettling S, Stamova S, Warta R, Schnolzer M, Rapp C, Rathinasamy A (2018). Identification of CRKII, CFL1, CNTN1, NME2, and TKT as Novel and Frequent T-Cell Targets in Human IDH-Mutant Glioma. Clin Cancer Res.

[35] Lee SY, Park YK, Yoon CH, Kim K, Kim KC (2019). Meta-analysis of gene expression profiles in long-term non-progressors infected with HIV-1. BMC Med Genomics.

[36] Schoppmeyer R, Zhao R, Cheng H, Hamed M, Liu C, Zhou X (2017). Human profilin 1 is a negative regulator of CTL mediated cell-killing and migration. Eur J Immunol.

[37] Salmond RJ, Filby A, Qureshi I, Caserta S, Zamoyska R (2009). T-cell receptor proximal signaling via the Src-family kinases, Lck and Fyn, influences T-cell activation, differentiation, and tolerance. Immunol Rev.

